# Diagnostic accuracy and acceptability of rapid HIV oral testing among adults attending an urban public health facility in Kampala, Uganda

**DOI:** 10.1371/journal.pone.0182050

**Published:** 2017-08-23

**Authors:** Joanita Nangendo, Ekwaro A. Obuku, Ismael Kawooya, John Mukisa, Annet Nalutaaya, Angella Musewa, Fred C. Semitala, Charles A. Karamagi, Joan N. Kalyango

**Affiliations:** 1 Clinical Epidemiology Unit, School of Medicine, College of Health Sciences, Makerere University, Kampala, Uganda; 2 Africa Centre for Systematic Reviews and Knowledge Translation, College of Health Sciences, Makerere University, Kampala, Uganda; 3 Faculty of Epidemiology and Population Health, London School of Hygiene and Tropical Medicine, London, United Kingdom; 4 Department of Internal Medicine, College of Health Sciences, Makerere University, Kampala, Uganda; 5 Makerere University Joint AIDS Program (MJAP), Kampala, Uganda; 6 Department of Pediatrics and Child Health, College of Health Sciences, Makerere University, Kampala, Uganda; 7 Department of Pharmacy, College of Health Sciences, Makerere University, Kampala, Uganda; Aga Khan University Hospital Nairobi, KENYA

## Abstract

**Background:**

The prevalence of HIV in Uganda is 7.3%, and yet nearly 40% of people living with HIV are unaware of their status. The current HIV testing policy which is strictly blood-based poses several challenges including: a need for high level laboratory skills, stringent waste disposal needs, and painful sample collection. It is envisaged that introduction of a rapid, painless HIV oral fluid test as a potential alternative is likely to increase the number of people testing. The aim of this study was to determine the diagnostic accuracy and acceptability of rapid HIV oral testing among adults attending Kisenyi Health Centre IV in Kampala.

**Methods and findings:**

We conducted a cross-sectional study among 440 adults recruited consecutively at Kisenyi Health Centre IV from January to March 2016. The diagnostic accuracy of the HIV oral test was assessed by comparing to the national HIV serial testing algorithm. We also assessed for acceptability among patients and health care workers (HCWs) by triangulating responses from a structured questionnaire, three focus group discussions and seven key informant interviews. Acceptability was defined as willingness to take the test at the time of the study and intention for future use of the test if it was availed.

The prevalence of HIV infection among study participants was 14.8%. The HIV oral fluid test was highly accurate with sensitivity of 100% (95% CI; 94.5–100.0), specificity of 100% (95% CI; 99.0–100.0), positive predictive value (PPV) of 100% (95% CI; 94.5–100.0) and negative predictive value (NPV) of 100% (95% CI; 99.0–100.0). Acceptability of HIV oral testing was also high at 87.0% (95% CI; 83.6–89.9). Participants preferred HIV oral testing because it was: pain free (91%, n = 399) and did not require blood draw (82%, n = 360).

**Conclusion:**

The HIV oral fluid test has high diagnostic accuracy and acceptability. HIV oral testing is a suitable addition to the national HIV testing strategies with the potential of increasing access to HIV testing services in Uganda.

## Introduction

HIV afflicts about 36.7 million people globally, and over 2.1 million new HIV infections were reported by the end of 2016 [1]. Sub-Saharan Africa (SSA) has the greatest burden where nearly one in every 25 adults is living with HIV, accounting for close to 70% of people living with HIV (PLHIV) globally [1]. In Uganda, the prevalence of HIV is at 7.3%, and over 120,000 new infections are diagnosed annually [2].

The Joint United Nations Program on HIV/AIDS (UNAIDS) set out to; identify 90% of PLHIV through HIV testing, initiate highly effective antiretroviral therapy (ART) in 90% of HIV positive individuals and attain undetectable HIV viral load in 90% of individuals on ART by 2020 [3]. However, only 55.8% of adults in Uganda have ever been tested for HIV, and nearly 40% of PLHIV are unaware of their status [2,4]. This low level of testing has been attributed to: poor access to HIV Counselling and Testing (HCT) services, fear of gossip, stigma and discrimination, as well as challenges in the process of HIV testing [5–8].

The current HIV testing algorithm in Uganda requires use of blood-based specimens, but that has challenges among both the patients and providers. The patient-related challenges include: fear of injections, discomfort and pain during blood sample draws, reduced privacy during testing, and social stigma. Among the providers, the challenges are: high level of skill required to carry out the test, risks of accidental exposure to infection and stringent waste disposal requirements [6–11].

Therefore introduction of a rapid HIV oral fluid test however, could potentially address challenges involved in blood-based testing [12]. Oral testing offers several additional benefits compared to blood-based testing. Such benefits include; convenience, user-friendliness and ability to extend testing boundaries beyond the conventional clinical settings [13–15]. In a non-clinical setting, oral testing also offers a unique opportunity of being delivered as a self-test which can stimulate testing among hard-to-reach sub-populations who would not otherwise test [16].

Currently, OraQuick^®^ Rapid HIV-1 antibody test (OraQuick^®)^ is the only second generation rapid oral swab test. It was approved to test for HIV using oral fluids by the United States of America (USA) Food and Drug Administration (FDA) in 2004 [17]. From systematic reviews and earlier research OraQuick^®^ has been reported to have high sensitivity and specificity each above 99% [18,19]. In addition, systematic reviews also suggest that HIV oral testing is highly acceptable to people in varying disciplines, socioeconomic status and locations [11,20]. Presently, the oral test is accessible for sale and public use in the USA and has markedly improved access to HCT services [21]. Some countries in sub Saharan Africa are also considering the introduction of HIV oral tests [22–24].

However, Uganda has not yet adopted non-conventional specimens like saliva for HIV testing despite the challenges associated with the existing blood-based testing. This is in part attributed to paucity of information on the feasibility and acceptability of HIV testing with non-conventional specimens which could be essential for effecting change in the current policy and practice on HTS in Uganda. We assessed the diagnostic accuracy and acceptability of rapid HIV oral testing among adults attending Kisenyi HCIV, a high volume public health facility in Kampala.

## Methods

### Ethics

We sought ethical approval to conduct this study from the Makerere University School of Medicine Research and Ethics Committee. We also obtained administrative permission from the Health Directorate of Kampala City Council Authority (KCCA), then the in-charge of Kisenyi Health Center IV (HC IV). All prospective participants provided informed consent (written or witnessed thumb print for the quantitative study and oral for the qualitative study). The study data were kept confidential, with access limited to only authorized personnel on the study team.

### Study design and setting

This was a cross-sectional study with quantitative and qualitative data collection methods from January to March 2016. The study was conducted at Kisenyi Health Centre IV (HC IV). Kisenyi HC IV is an urban clinic intended to serve about 100,000 people within the health sub-district, and it is administered by Kampala Capital City Authority (KCCA) (the regulatory body for Uganda’s capital city). The Health Centre offers a wide range of services including: inpatient services, emergency care, and outpatient services like; integrated TB/HIV prevention, care and treatment and health education. It serves a population of mainly traders within the central business district, as well as slum dwellers from the neighborhood. The prevalence of HIV in Kampala is estimated at 7.1% [2].

### Study participants

The study included all adults (aged ≥ 18 years) who sought HCT services from Kisenyi HCIV outpatients department during the study period and gave informed consent to participate in the study. We excluded people who were taking ART, could not speak or comprehend English, Luganda, or Swahili languages (the most commonly spoken languages in the region where the study was conducted) or who were too sick to participate.

### Sampling and sample size

We consecutively enrolled a total of 440 participants. The sample size for HIV oral test diagnostic accuracy was determined using the modified Kish Leslie formula for diagnostic studies [25]. We assumed a 95% confidence level (CI), sensitivity of 99.7%, specificity of 99.9% [18], and HIV prevalence of 7.1% [2]. Sensitivity and specificity were obtained from a report of four studies conducted by The Centre for Disease Control and Prevention (CDC) in diverse settings in USA. One of the studies was among pregnant women undergoing HIV screening at 18 hospitals in six USA cities as part of the Mother–Infant Rapid Intervention at Delivery (MIRIAD) study while the other three were among high-risk persons at 41 community outreach sites in Minneapolis, Minnesota and three HIV testing sites and two sexually transmitted disease (STD) clinics in Los Angeles, California and Phoenix, Arizona [18]. Prevalence of HIV was extracted from the Uganda AIDS Indicator survey (UAIS) [2]. For acceptability, the sample size was obtained using the formula for single proportions [26]. We assumed a 95% CI, total width of the confidence interval of 0.1, a non-response rate of 10% and acceptability of OraQuick^®^ of 0.468 (as reported in a study in Mozambique) [27].

### Quantitative data collection

The data were collected by research assistants who were experienced HIV counselors and laboratory technologists. The research assistants were trained for two days on the study procedures. The HIV tests (blood-based and oral fluid) were conducted in parallel and performed according to the manufacturer’s instructions by two independent laboratory technologists, who worked from separate rooms and submitted test results separately. After that, a third independent laboratory technologist cross-checked the blood and oral test results for any discrepancies. For any disagreements between the tests, the independent laboratory technologist repeated the tests in line with the national serial algorithm. A counselor then informed the respective participant of the test results after post-test counseling.

To perform the HIV oral test, each participant collected an oral fluid sample by gently swabbing the flat pad of OraQuick^®^ once across the outer gums (upper and lower) under the direct supervision of a technologist. The technologist then placed the pad in a vial with a pre-measured amount of developer and results were read after 20 minutes. A blood sample was also obtained by finger stick for rapid diagnostic tests following the national serial testing algorithm. The national serial algorithm involves using three rapid diagnostic tests; Determine^®^ to screen, then Stapak^®^ as a confirmatory test and lastly Unigold^®^ as a tie breaker in case of discrepancies between the first two [28]. After testing, a structured questionnaire (S1 File) was administered to each participant to capture more information on acceptability of the oral fluid test. All clients that tested HIV positive were immediately linked to the ART clinic in Kisenyi HCIV.

### Qualitative data collection

The qualitative study was carried out to explain the quantitative findings on acceptability of rapid oral testing. The study participants included both the clients and HCWs at Kisenyi HC IV. For the focus group discussions (FGDs), clients were selected by snow ball sampling from amongst those who had taken the oral fluid test earlier. One person (the first participant) identified by study team helped to invite others to join the discussions. Three FGDs (two with men and one with women) were held, moderated by a trained interviewer using a topic guide (S2 File). Seven key informant interviews (KIIs) were also held with HCWs from Kisenyi HC IV and from other HIV/AIDS related treatment and research centres. The cadres of the HCWs interviewed included a laboratory technologist, a nurse, the HCT-coordinator and counselor (all based at Kisenyi HC IV), the supervisor clinic services at the AIDS Information Centre in Kisenyi, and two senior researchers in HIV/AIDS from Makerere-Mbarara Joint AIDS Program (MJAP) and Makerere University School of Public health. All the interviews were aimed at obtaining the provider’s perspective to HIV oral testing and were recorded using an audio recorder.

### Quantitative data analysis

Quantitative data was analyzed using Stata version 13.0 (StataCorp, College Station, TX, USA). Diagnostic accuracy was measured as sensitivity, specificity, negative predictive value (NPV) and positive predictive value (PPV) their 95% CI. Acceptability (defined as: willingness to use versus unwillingness to use the test [29], eagerness to recommend the test to someone else, and positive thoughts or attributes on the test for example convenience and time to results [30]) was measured using proportions and their 95% CI. Other variables like: socio-demographic factors; HIV testing histories, HIV-related risky behaviors, health care procedures, and experiences from HIV testing were also descriptively analyzed to define the study population.

### Qualitative data analysis

All audio recordings obtained from the FGDs and KIIs were transcribed verbatim and analyzed thematically following prior themes from similar studies [8,11,15,27,31]. The themes included: pain free procedure, no blood drawn, requires short time to results, offering a good degree of privacy and convenience [11,15,27,31]. We triangulated the findings on acceptability of HIV oral testing from quantitative and qualitative results.

## Results

### Description of the study population

A total of 440 participants with a median age of 30 years were enrolled into the study during the study period. About half of participants were males 51.4% (226/440), 52.7% (232/440) were married and about 71% (311/440) were self-employed (Table 1).

**Table 1:** Socio-demographic characteristics of study participants at Kisenyi Health Centre IV, January to March 2016.

Variable	Measure
**Median age,** (min, max)	30 (18, 62)
**Male,** n (%)	226 (51.4)
**Religion,** n (%)	
Protestant	147 (33.4)
Catholic	145 (33)
Moslem	85 (19.3)
Born-again & Others*	63 (14.3)
**Ugandan nationality,** n (%)	429 (97.5)
**Marital status,** n (%)	
Single	84 (19.1)
Married/Living together	232 (52.7)
Never married	71 (16.1)
Divorced/Separated	53 (12.1)
**Education level,** n (%)	
No formal education	20 (4.5)
Primary	161 (36.6)
Ordinary level secondary	151 (34.3)
Advanced level secondary	42 (9.6)
Tertiary & University	66 (15)
**Occupation,** n (%)	
Professional job	68 (15.5)
Un-employed	61 (13.8)
Self-employed	311 (70.7)

*Seventh day Adventist (2/440), Jehovah's witness (2/440) & Rastafarian (1/440)

Nearly 40% (177/440) of the participants had risks of exposure to HIV infection at work, 82% (361/440) had ever tested for HIV and 76% (332/440) of them had tested within the preceding year (Table 2).

**Table 2:** HIV testing history among 440 study participants at Kisenyi Health Centre IV, January to March 2016.

Variable	Measure
**Risk of exposure at work (Yes),** n (%)	177 (40.2)
**Ever taken an HIV test (Yes),** n (%)	361 (82.1)
**Time of last HIV test,** n (%)	
2 or more years	29 (6.6)
Less than or equal to 1 year	332 (75.5)
Never tested	79 (17.9)
**Result of last HIV test (Positive)** **^+^**, n (%)	13 (3.6)
**Knowledge of partner's HIV status (Yes),** n (%)	224 (50.9)
**Partner's HIV status (Positive)** *, n (%)	23 (10.3)

^+^ Among those who have ever tested for HIV (N = 361)

* Among those who know their partner(s) HIV status (N = 224)

About 15% (68/440) of participants had multiple sexual partners, 28% (121/434) consumed alcohol, and of those who were not married, about 53.4% (111/208) never used condoms during sexual intercourse (Table 3).

**Table 3:** HIV risky behaviors among study participants at Kisenyi Health Centre IV, January to March 2016.

Variable	Measure
**Present number of sexual partners,** n (%)	
None	75 (17.1)
One	297 (67.5)
2 or more	68 (15.4)
**Consistent condom use with non-marital partners**^**^+^**^, n (%)	
Sometimes	30 (14.4)
Always	67 (32.2)
No	111 (53.4)
**Current involvement in sex trade (Yes),** n (%)	24 (5.5)
**Alcohol use (Yes)** ^3^*, n (%)	121 (27.9)
**Drug use (Yes)** ^3^*, n (%)	11 (2.5)

^+^ N = 208

*missing data on the variable: Alcohol use (6/440), Drug use (1/440)

### Diagnostic accuracy of the HIV oral test

Among the study participants, the prevalence of HIV infection was 14.8% (95% CI; 11.6–18.4). From each algorithm, HIV test results were concordant; 65 positives were recorded. There were no discrepancies between all test results. The diagnostic accuracy parameters of OraQuick^®^ were; sensitivity 100% (95% CI; 94.5–100.0), specificity 100% (95% CI; 99.0–100.0), PPV 100% (95% CI; 94.5–100.0) and NPV 100% (95% CI; 99.0–100.0) respectively (Table 4).

**Table 4:** Diagnostic accuracy of OraQuick® Rapid HIV-1 antibody test compared to HIV infected^1^, N = 440.

Parameter	Value
Sensitivity (95% CI)	100% (94.5%– 100.0%)
Specificity (95% CI)	100% (99.0%– 100.0%)
Positive Predictive Value (95% CI)	100% (94.5%– 100.0%)
Negative Predictive Value (95% CI)	100% (99.0%– 100.0%)

^**1**^ HIV infected as defined by both a positive Determine® and Stapak® test result

### Acceptability of HIV oral testing

From the quantitative results, acceptability of HIV oral testing was 87.0% (95% CI; 83.6–89.9) (Table 5).

**Table 5:** Acceptability of HIV oral testing among adults at Kisenyi Health Centre IV from January to March, 2016.

Variable	Measure
Awareness about HIV Oral test*, n (%)	
No	376 (85.7)
Yes	63 (14.3)
**Willing to use the HIV Oral test,** n (%)	
Yes	390 (88.6)
No	50 (11.4)
Intention to use the HIV Oral test*, n (%)	
Yes	397 (90.4)
No	42 (9.6)
Overall acceptability^+^, n (%)	
Yes	383 (87)
No	57 (13)

*missing data on the variable: Awareness of HIV oral test (1/440), Intention to use the HIV oral test (1/440)

**^+^**acceptability measured as a single outcome variable generated from willingness- and intention- to use the HIV oral test

The reasons given for the acceptability included: being pain free (91%, 399/440), requiring no blood to be drawn (82%, 360/440), having an easy process of sample collection (58%, 257/440), convenience (41%, 180/440) and enabling frequent testing (28%, 124/440) (Table 6).

**Table 6:** Reasons for acceptability of HIV oral testing among adults at Kisenyi Health Centre IV from January to March, 2016.

Variable	Measure
Reasons to prefer HIV oral testing*, n (%)	
Pain free	399 (90.7)
No blood drawn	360 (81.8)
Short time to results	51 (11.6)
Privacy	19 (4.3)
Non-invasive	16 (3.6)
Reliable	37 (8.4)
Easy sample collection	257 (58.4)
Convenient	180 (40.9)
Enable frequent testing	124 (28.2)
Reasons not to prefer HIV oral testing*, n (%)	
Not sure of the accuracy	30 (6.8)
Prefer blood tests	22 (5)
Don't want new tests	4 (0.9)
Confident in blood-based testing	7 (1.6)
No reason	264 (60)

* The reasons were assessed using multiple choice questions

These findings were similar to those seen from the subsequent qualitative data as highlighted below;

It is a painless test.

*“It does not create pain to the patient compared to pricking to get a blood sample*. *Some of these clients do not come to test because they fear to be pricked because of the pain involved but with saliva you do not experience any pain so you find anyone willing to do the test”*, (KII).

It is simple, easy to use and time-saving.

*“… It is very simple*. *We just come*, *they open the test kit*, *then you swab your mouth where there is saliva and then you sit to wait for the results*. *It is such a wonderful procedure …compared with the other one with blood*, *I feel it is very comfortable*, *it is very convenient”*, (FGD men).

HIV oral testing offers privacy and confidentiality.

*“… I can even do it from my office here now …*.. *for the blood test*, *you have to go through the counseling even if you know what to do there is no privacy you have to go through all the phases pretest and post-test counseling but for this one I just do it and I can take it home and give to my husband without bringing in the third party to test it for himself”*, (KII).

HIV oral testing is reliable

*“We compared blood test and rapid HIV oral fluid test; the results were tallying like if it came positive using the blood it also comes positive using the saliva like it was you know it was something very interesting”*, (KII).

Other perceptions to HIV oral testing included;

It poses no risk of exposure to HIV infection.

*“Aaa I think this method is better in that it cannot expose the health worker to get infected like for the use of blood because the use of blood you can easily get in touch with blood”*, (FGD Men).

HIV oral testing could increase uptake of rapid HCT services.

*“…Those who fear to test*, *fear to shade blood will embrace this method since this is just acquiring saliva it will be simple for them*. *This is good*, *many people will come out to test for HIV because the procedure is painless and easy”*, (FGD Women).

*“It will also encourage people that cannot access such services at the centers to be tested even in their homes it doesn’t even need a lot of expertise the moment people go through the training they can”*, (KII).

Besides the positive perceptions to HIV oral testing, participants also perceived a potential drawback that if offered as a self-test, HIV oral testing might increase potential for self-harm especially in case of HIV positive test results.

*“I think this method somehow is good*, *on the other hand you see people still need counseling*. *Now when you bring saliva whereby somebody is able to do it alone*, *what if that client is positive how is it going to happen*? *You might increase people hanging themselves because of stigma in the community*. *We need to know the way forward before it is brought on board so that at the end if we are to start using*, *we are counseled first like the in normal procedure”*, (FGD Men).

## Discussion

With sensitivity, specificity, and predictive values at 100.0%, the HIV oral test was comparable to the current national standard of HIV diagnosis in our study. Above all, the HIV oral test was acceptable to 87.0% of all the participants approached by the study team. The clients and HCWs had similar perceptions which included; painlessness, convenience, having no need for blood draw, taking short time to obtaining results and ability to give a good degree of privacy. These properties are important to promote for the use of the oral test, and spur an increase in HIV testing towards identifying 90% of people with HIV.

Our results are in agreement with earlier studies done in Africa and elsewhere in the world. A study in Zimbabwe showed sensitivity 100% (95% CI; 98.0–100.0) and specificity 99.7% (95% CI; 98.4–99.9), another study in Zambia showed a sensitivity of 98.7% (95% CI; 97.5–99.4) and specificity of 99.8% (95% CI; 99.6–99.9) [15,32]. In Malawi, sensitivity was 97.9% (95% CI; 87.9–100) and specificity of 100% (95% CI; 97.8–100), while in Mozambique the sensitivity was 99.8% (95% CI; 98.7–99.9) and specificity was 99.8% (95% CI; 99.3–99.9) [27,31]. Additionally, in the systematic review by Pant et al, from the supervised and unsupervised strategies, the HIV oral test had specificity from 99.8% to 100% and sensitivity from 92.9% to 100% [11]. These studies reinforce our results that the HIV oral test could be a suitable complement to the current national HIV testing algorithm.

Of concern however, the high accuracy of the HIV oral test reported in our study could be attributed to the fact that all testing was done under direct supervision of trained personnel which minimized operator errors during test performance and result interpretation [11]. Nonetheless, the sensitivity of the oral test can be lower because oral fluids have lower antibody concentrations than blood-based specimens thereby creating a possibility of obtaining false negative results especially in sero-converting patients [10,16,33]. False negative results are a potential drawback to the application of the HIV oral test. It is important to provide users with adequate information to perform the test correctly, and how to take appropriate action in case of a negative result.

Our findings also showed that the HIV test was highly acceptable, consistent with Pant et al who reported an acceptability range of 74% to 96% [11]. In this systematic review, preference of the HIV oral test was mainly due to attributes of: non-invasiveness, confidentiality, privacy and anonymity, convenience, speed, time efficiency and potential to; increase testing frequency and provide a sense of empowerment [11]. A study from Mozambique also reported less pain and greater ease of use as the major reasons for expressing interest in HIV oral testing [27].

Furthermore, our findings suggest that participants had high regard for the HIV oral test and would embrace it if included in the national HIV testing programs. Noteworthy; however, is that privacy (4.3%) was not a major issue, implying that delivery of the HIV oral test under supervision would be feasible. So, oral testing could offer a viable testing option to individuals who would otherwise not test for HIV using the blood-based algorithm [31].

Nevertheless, our study also revealed some of the possible negative aspects of the HIV oral test like; fear of self-harm and doubts about accuracy of test results. Fear of self-harm would be a potential barrier to the uptake of HIV oral testing especially if delivered as a self-test. Since testing is likely to occur in the absence of proficient pre- and post-test counseling, clients might be left helpless especially if faced with unclear or surprising testing outcomes. The fear of self-harm has also been reported in previous research [11,24,27]. In Mozambique, the priority for reliability in HIV testing was accorded to whole blood testing [27], while Pant et al mentioned doubts about accuracy and potential misuse of oral kits as probable hindrances [11]. These fears raise concerns about the feasibility of HIV oral testing hence require that prior to population-wide roll-out, appropriate measures are taken to counter such possible uncertainties.

Our study had some limitations. It was conducted in a clinical setting among an urban population with high HIV prevalence. This population may differ from the general population in Uganda which is predominantly rural, with poorer health seeking behaviors but lower HIV prevalence [2]. The study population may also not be representative of the most desired target for an oral fluid test since over 80% of the participants had ever tested for HIV and seemed relatively comfortable with their test results. We also used rapid diagnostic tests as a reference standard but this is less than the ideal (DNA PCR). So, though not encountered in our study, rapid tests have been reported to pose a risk of false negatives especially in acute HIV infection before sero-conversion as well as in patients already on ART with very low CD4 counts. Similarly, there can also be a risk for false positives for instance during autoimmune diseases, viral and parasitic infections, and malignancies like lymphoma [34,35]. Finally, triangulation of quantitative and qualitative data on acceptability could also have compromised validity of the results due to inherent difficulties of representing a lived experience through text and numbers.

Nevertheless, our findings add to the evidence that HIV oral testing would be a potential approach for increasing access to HCT services. Even though Uganda’s policy on HCT is still tailored to only blood-based HIV testing, these findings could guide considerations on the use of non-conventional specimens in HCT.

## Conclusion

The HIV oral test has high diagnostic accuracy and is very acceptable among clinic attendees and health care workers in Kampala, Uganda. Introduction of HIV oral testing should be considered so as to complement the existing HIV testing methods in Uganda.

## Supporting information

S1 File This is the HIV oral test questionnaires- FINAL.pdf. (PDF)

S2 File This is the HIV oral test topic guide.pdf. (PDF)
