# Supplementary material for: Diagnostic accuracy and acceptability of rapid HIV oral testing among adults attending an urban public health facility in Kampala, Uganda
Source: PLoS One. 2017 Aug 23;12(8):e0182050. doi: 10.1371/journal.pone.0182050 (PMC5568333; doi:10.1371/journal.pone.0182050)
Supplement: S1 File — (PDF) [file pone.0182050.s001.pdf]

Participant screening log

Study Title: Performance, acceptability and the associated factors of HIV oral testing among adults attending Kisenyi Health Centre IV in Kampala

Investigator: ____________________________ (initials)

Participant ID #: _______________ Date: ___/___/_____

ELIGIBILITY CRITERIA CHECKLIST

INCLUSION	EXCLUSION
All boxes must be checked **Yes** for Participant to be considered eligible	All boxes must be checked **No** for Participant to be considered eligible
**Yes** **No**	**Yes** **No**
☐ ☐ Age; (18 years and above)	☐ ☐ Is very ill
☐ ☐ Attending Out- Patient Department	☐ ☐ Unable to comprehend either English, Luganda or Kiswahili
☐ ☐ Seeking HCT services between January and March 2016	☐ ☐ Is HIV-positive and already taking ARVs

I have reviewed this Participant's medical information and have determined that they have met the criteria for eligibility into the above-mentioned study.

Completed by ____________________________ ___/___/______ __________

Person Verifying Eligibility Date Time

Information Reviewed by ____________________________ ___/___/______

Principal Investigator Date

Study questionnaire in English

A QUESTIONNAIRE TO INVESTIGATE THE ACCEPTABILITY AND ASSOCIATED FACTORS OF HIV ORAL TESTING AMONG ADULTS IN KAMPALA

Study ID NO___________ Date of interview: _____/_____/_____

A. <u>Socio-Demographic characteristics</u>

1. Date of Birth:____/____/_______ (dd/mm/yyyy)
2. Age (in complete years):____________
3. Sex: ☐ 1=Male ☐2=Female
4. Religion: ☐1=Protestant ☐2=Catholic ☐3=Moslem
   ☐4= born-again ☐5=others (specify) ________________
5. Ethnicity: ☐1= Baganda ☐2= Banyankole ☐3=Basoga
   ☐4= Bakiga ☐5= Somali ☐6= others (specify) ____________
6. Nationality: ☐1= Ugandan ☐2= Kenyan ☐3= Tanzanian
   ☐4= Somali ☐5= Southern Sudanese ☐6= others (specify) ___________
7. Marital status: ☐1=Single ☐2=Married/Living together ☐3= Never married
   ☐4. Widowed ☐5=Divorced/Separated ☐6=others (specify) __________
8. Highest level of education: ☐1=No formal education ☐2=Primary ☐3=S1-S4
   ☐4=S5-S6 ☐5=Tertiary ☐6=University
9. Occupation: ☐1=Un-employed ☐2=Self-employed (specify)______________
   ☐3= professional job (specify)___________________

B. Health care procedures

10. Have you undergone surgery in the past three months? ☐1=Yes ☐2=No

11. Have you had blood transfusion in the past three months? ☐1=Yes ☐2=No

12. At your job, do you make contact with human body fluids or used sharps like needles?

    ☐1=Yes ☐2=No

C. <u>HIV testing history</u>

13. Have you ever tested for HIV? ☐1 = Yes ☐2 = No *<u>(If No skip to 17)</u>*

14. When was your last HIV test?

    ☐1 = 1-3 months ☐2= 4-6 months

    ☐3=7-9 months ☐4= one year ago ☐5=More than 2 years ago

15. What were the results from your last HIV test? ☐1=Positive ☐2=Negative

16. How often do you get tested for HIV?

    ☐1 = I have never been tested ☐2= Once in a year

    ☐3=1 or 2 times in a year ☐4= 3 or 4 times in a year ☐5=More than 4 times in a year

D. <u>HIV-related risky behaviors</u>

17. How many sexual partners have you had in the last 6 months? (If you are uncertain, you can estimate.) |___|___|

18. Presently, how many sexual partners do you still have? |___|___|

19. Do you know your partner’(s) HIV status? ☐1=Yes ☐2=No ☐3=Don’t know *(If No skip to 21)*

20. If yes, what is your partner(s) HIV status? ☐1=Positive ☐2=Negative ☐3=Don't know

21. Do you always use a condom when having sexual intercourse?

☐1=Yes ☐2=No ☐3= Sometimes

22. Have you ever traded sex for money, housing, drugs, protection, or other goods?

☐1=Yes ☐2=No

E. Alcohol and drug use

23. How often do you take an alcoholic drink? *(If 1, skip Qn.29)*

☐1 = I don't drink anything alcoholic ☐2= Once a week

☐3= 2-4 times a week ☐4= every day of the week ☐5= Once a month

24. Which type of alcoholic drink do you usually take?

☐1= Wine ☐2= Beer

☐3= Spirits/waragi/gin/whisky/vodka ☐4= Local brew (specify) ______________

25. At what time of the day do you usually take the alcoholic drink?

☐1= Early in the morning ☐2= during my lunch break

☐3= in the evening after work ☐4= All day as I work

27. Are you more likely to engage in sexual intercourse after taking an alcoholic drink?

☐1= Yes ☐2= No ☐3= don't know

28. Even when you have taken an alcoholic drink still use a condom? ☐1=Yes ☐2=No

29. Do you use any intoxicants? ☐1=Yes ☐2=No *(If No skip to Qn 32)*

30. Which intoxicants do you use?

☐1 = Marijuana/ Shisha ☐2= Khat ☐3= Petrol

☐4= Kubba ☐5= others (specify) ___________

31. How often do you use the intoxicants?

☐1 = Once a week ☐2= 2-4 times a week

☐3= every day of the week ☐4= Once a month

F. Perceptions on the HIV oral test

32. Have you ever heard about the rapid HIV oral fluid test? ☐1=Yes ☐2=No

33. Would you ever consider testing with the HIV oral fluid test in future?

☐1=Yes ☐2=No ☐2=Not sure

34. Would you be more likely to test if this HIV oral testing service is availed to you?

☐1= Yes ☐2= No ☐3= don't know

35. Why would you prefer to test using HIV oral testing? ***(Tick all possible options) (Probe the participant)***

☐1= Pain free procedure ☐2= No blood drawn ☐3= requires short time to results

☐4= Offers a good degree of privacy ☐5= Non-invasive ☐6= It is reliable

☐7= Ease of sample collection ☐8= Convenience ☐9= I can be able to test more often

☐10= others (specify) ___________

36. Why would you not consider testing with the HIV oral fluid test? ***(Tick all possible options) (Probe the participant)***

☐1= I have never heard of this test ☐2= I am not sure about its accuracy

☐3= I just prefer the blood test ☐4= I don't want to test with new methods

☐5= I am more confident if tested using blood ☐6= It is unreliable ☐7= others (specify)

Results of laboratory tests ***(To be filled by the counselor)***

37. HIV sero status from rapid blood test ☐ 1=negative ☐ 2=positive

38. HIV sero status from rapid Oral test ☐ 1=negative ☐ 2=positive

39. Are the test results concordant? ☐ 1=Yes ☐ 2=No

(To be filled only after repeat testing on the serial blood algorithm)

40. What is the result of the HIV test? ☐ 1=negative ☐ 2=positive

Study questionnaire in Luganda

A QUESTIONNAIRE TO INVESTIGATE THE ACCEPTABILITY AND ASSOCIATED FACTORS OF HIV ORAL TESTING AMONG ADULTS IN KAMPALA

Study ID NO___________ Ennaku zo'mwezi: _____/_____/_____

A. Ebikwata kubulamu bwo

1. Wazalibwaddi?____/____/_______ (dd/mm/yyyy)
2. Oyina emyaka emeka (emijuvu)? ____________
3. Obutonde? ☐ 1=Mwami ☐2=Mukyala
4. Oli wa nzikiriza ki?

   ☐1=Mukurisitayo ☐2=Mukaturiki ☐3=Musiramu ☐4= Mulokole

   ☐5= Endala (nzikiriza ki?) ________________
5. Oliwa gwanga ki?

   ☐1= Muganda ☐2= Munyankole ☐3=Musoga ☐4= Mukiga ☐5= Musomali

   ☐6= Edala (gwanga ki?) ____________
6. Oli mutuuze wansi ki?

   ☐1= Uganda ☐2= Kenya ☐3= Tanzania ☐4= Somalia

   ☐5= Sudan eyamaserengeta ☐6 = Edala (nsi ki?) ___________
7. Oli mufumbo?

   ☐1=Ndi nzeka ☐2=mufumbo/mubrera mwembi ☐3= Sifumbirangako

☐4. Namwandu/Ssemwandu ☐5=twayawukana/nanoba ☐6=Ebilala (nga ki?) __________

8. Wasoma kutuuka wa?

☐1=Sisomangako ☐2=Mukisooka pakaku kyomusanvu ☐3=Siniya esooka pakaku yokuna ☐4= Siniya eyokutano pakaku yomukaaga ☐5= Mu tekiniko ☐6=Yunivasite

9. Okola mirimu ki? ☐1=Sikola ☐2=Nekozesa (murimu ki?)______________

☐3= gwa buyigirize (murimu ki?)______________

B. Emirimu egyobusawo

10. Walongoosebwako mu myezi esatu egiyise? ☐1= Yee ☐2=Nedda

11. Watekebwako omusayi mu myezi esatu egiyise? ☐1= Yee ☐2= Nedda

12. Omurimu gwo gulimu okukola no musaayi, ebiwundu, oba okulongosa abantu oba ebifumita ngempiso oba obwambe? ☐1= Yee ☐2= Nedda

C. Ebikwata kukwekebeza akawuuka kamukenenya

13. Wali wekebeza akawuuka kamukenenya? ☐1 = Yee ☐2 = Nedda *(If Nedda skip to 17)*

14. Wasemba ddi okwekebeza akawuuka kamunekenenya?

☐1 = emyezi 1-3 ☐2= emyezi 4-6 ☐3= emyezi 7-9

☐4= omwaka gumu emabega ☐5=myaka ebiri nokusingawo emabega

15. Alipoota gyewafuna mukwekebeza akawuuka kamukenenya okwasembayo yali etya?

☐1=Nasangibwa nakawuuka ☐2= Ssasangibwa nakawuuka

16. Oteraddi okwekebeza akawuuka kamukenenya?

☐1 = Sekebezangako ☐2= Omurundi gumu mumwaka ☐3= Omurundi gumu oba ebiri mumwaka ☐4= Emurundi esatu oba ena mumwaka ☐5= Emurundi ena nokusoba muwaka

D. Ebikwata ku mpisa ezobukaba

17. Mumyezi omukaaga egiyise, obabbe nabaganzi bamekka? (Bwoba tewekakasa, oyinza okutebereza). |___|___|

18. Ennakuzino, abaganzi okyalina bameka? |___|___|

19. Omanyi embeera yamuganziwo oba bangazibo kukawuuka kamukenenya?

☐1 = Yee ☐2 = Nedda ☐3 = Simanyi *(If Nedda skip to 21)*

20. Bwoba omanyi, muganziwo oba bangazibo ayimiridde atya/bayimiridde batya kukawuuka kamukenenya?

☐1=Alina/Balina kawuuka ☐2= Talina/Tebalina kawuuka ☐3 = Simanyi

21. Buli lwewegata nemuganziwo/nebaganzibo, okozesa/mukozesa akapiira/obupiira bukalimpitawa? ☐1 = Yee ☐2 = Nedda ☐3 = Oluusi

22. Wali wegaase nomuntu olwokufuna sente, ewokusula, eddagala, obukuumi, oba ekintu ekirala kyona? ☐1 = Yee ☐2 = Nedda

E. Ebikwata ku kunywa ebitamiiza nokukozesa ebilagalagala

23. Oteraddi okunywa kubitamiiza? *(If 1, skip Qn.29)*

☐1 = Sinywa kintu kyona kitamiiza ☐2= Omurundi gumu muwiiki ☐3= Emirundi ebiri oba ena muwiiki ☐4= Bulu lunaku lwa wiiki ☐5= Omurundi gumu mumwezi

24. Bitamiizaki kika kyi byoteera okunywa?

☐1= Wayini ☐2= Biya

☐3= Waragi/gin/whisky/vodka ☐4= Omwenge omuganda (kika ki?) _____________

25. Ebitamiiza otera kubinywa mubiseera ki mulunaku?

☐1= Kumakyanyo ☐2= musaawa zekyemisana

☐4= akawungeezi nga mazze okukola ☐2= Olunaku lwona nga bwenkola

26. Oba onywedde ebitamiiza, kyongeza obwagazibwo okwegata?

☐1= Sinywa kintu kyona kitamiiza ☐2= Yee ☐3= Nedda ☐4= Simanyi

27. Waddenga onywedde ebitamiiza, era okozesa akapiira kalimpitawa oba ogenda okwegata?

☐1= Yee ☐2= Nedda ☐3= Simanyi

28. Okozesa ku bilagalalagala? ☐1= Yee ☐2= Nedda *(If Nedda skip to Qn 32)*

29. Bilagalalagala ki byokozesa?

☐1 = Enjaga/ Shisha ☐2= Khat ☐3= Amafuuta gemmotoka

☐4= Kubba ☐5= Ebirala (ngaki?) ___________

31. Oteraddi okukozesa bilagalalagala?

☐1 = Omurundi gumu muwiiki ☐2= Emirundi ebiri oba ena muwiiki

☐3= Bulu lunaku lwa wiiki ☐4= Omurundi gumu mumwezi

F. Ebirowoozo kunkola eyokukozesa amalusu okukebera akawuuka kamukenenya

32. Wali owulide kunkola eyokukebera akawuuka kamukenenya ngo'kozesa amaluusu?

☐1= Yee ☐2= Nedda

33. Gyeebuuja mumaaso singa enkola eno eba eleteeddwa m'Uganda, oyinza okulondawo oba okusalawo okugyikozesa okwekebezesa akawuuka kamukenenya?

☐1=Yee ☐2=Nedda ☐3=Sseekakasa

34. Olowooza emirundi gyewekebeza akawuuka kamekenenya gyiyinza okweyongera singa enkola eyokukebezesa amaluusu eletebwa m'Uganda?

☐1= Yee ☐2= Nedda ☐3= Simanyi

35. Olowooza songa ki eziyinza okukwagazisa enkola eyokwekebera akawuuka kamukenenya okuyita mu maluusu? ***(Londako zona zolowooza) (yogereza participant).***

☐1= Tereeta bulumi ☐2= Teyetaaga ku kujako musaayi ☐3= Etwala akaseera katono

☐4= Esobola okukolebwa mu kyama ☐5= Enkola teringiriza nyo bulamu ☐6= Enkola yesigika ☐7= okufuna amaluusu kyangu nyo ☐8= Enkola nyangu nyo

☐9= Osobola okwekebeza emirundi mingi okusingako egyabulijjo

☐10= Ebirala (ngaki?) ___________

36. Olowooza songa ki eziyinga okukulemesa kokuzesa enkola eyokwekebera akawuuka kamukenenya okuyita mu maluusu? ***(Londako zona zolowooza) (yogereza participant).***

☐1= Eno enkola sijiwuulirangako ☐2= Sesiga butuufu bwankola eno

☐3= Nsinga kwagala kwekebeza namusaayi ☐4= Sagala kwekebezesa nankola mpya

☐5= Mba mugumu singa nekebeza ngankozesa omusaayi ☐6= Enkola sigyesiga

☐7= Ebirala (ngaki?) ___________

Alipoota zokwekebeza akawuuka kamukenenya *(Zakujuzibwamu kansala).*

37. Alipoota yokukebera akawuuka kamukenenya okuyita mu musaayi

☐1=Alina kawuuka ☐2= Talina kawuuka

38. Alipoota yokukebera akawuuka kamukenenya okuyita mu maluusu

☐1=Alina kawuuka ☐2= Talina kawuuka

39. Aliipota zo kukebera akawuuka kamukenenya okuyita mu musaayi ne mu maluusu zifaanagana? ☐ 1=Yee ☐ 2=Nedda

(Wakujuzibwa nga tumazze okuddamu okukebera akawuuka kamukenenya nga tukozesa omusaayi)

40. Aliipota esembayo ekwatagana ku kukebera akawuuka kamukenyenya eri etya?

☐ 1= Alina kawuuka ☐ 2= Talina kawuuka

Study questionnaire in Kiswahili

TAFSIRI YA MASWALI

Orodha ya maswali ya utafiti kuchunguza kukubalika pamoja na masuala yanayohusika na kupimwa kwa vvu kutumia njia ya mdomo miongoni mwa watu wazima wanaohudhuria kituo cha afya ya kisenyi kilicho kampala

Nambari ya itifaki: ___________ Tarehe ya hoji: _____/_____/_____

A. Sifa za kijamii ya Watu

1. Tarehe ya Kuzaliwa:____/____/_______ (dd/mm/yyyy)
2. Umri (kwa miaka):____________
3. Jinsia: ☐ 1= kiume ☐2= kike
4. Dini: ☐1= Kiprotestanti ☐2= Katoliki ☐3= Kiislamu

   ☐4= Kuokoka ☐5= Nyinginezo (taja) _________________
5. Ukabila: ☐1= Baganda ☐2= Banyankole ☐3=Basoga

   ☐4= Bakiga ☐5= Somali ☐6= Nyinginezo (taja) ____________
6. Uraia: ☐1= Muyuganda ☐2= Mkenya ☐3= Mtanzania

   ☐4= Msomali ☐5= Msudan Kusini ☐6= Nyinginezo (taja) ___________
7. Hali ya ndoa: ☐1= Bila kuolewa ☐2= Oa au kuishi pamoja ☐3=Kamwe sijaoa/kuolewa

   ☐4. Mjane ☐5= Waliotaliki/Tenganishwa ☐6= Nyinginezo (taja) __________
8. Ngazi ya juu ya elimu: ☐1= Hakuna elimu rasmi ☐2= chuo cha Msingi ☐3=S1-S4

   ☐4=S5-S6 ☐5= chuo cha kati ☐6= Chuo Kikuu

9. Kazi: ☐1= Kutoajiriwa ☐2= Kujiajiri (taja) ______________

☐3= kazi kitaalamu (taja)___________________

B. Taratibu za matibabu

10. Je, Kwa miezi mitatu iliyopita, umewahi kupitia upasuaji? ☐1= Ndio ☐2= La

11. Je, umewahi kuongezewa damu katika kipindi cha miezi mitatu iliyopita?

☐1= Ndio ☐2= La

12. Katika pilka pilka ya kazi yako, je, unapatana na sampuli za bina damu au kutumia vifaa kama sindano? ☐1= Ndio ☐2= La

C. Historia ya kupimwa VVU

13. Umewahi kupimwa VVU? *(If No skip to 17)*

☐1 = Ndio ☐2 = La

14. Wakati ilikuwa ni mtihani wako wa mwisho VVU?

☐1 = miezi 1-3 ☐2= miezi 4-6 ☐3=miezi 7-9

☐4= mwaka mmoja uliopita ☐5= Zaidi ya miaka 2 iliyopita

15. Ni nini ilikuwa matokeo ya kipimo chako cha VVU ya mwisho?

☐1= Chanya ☐2= Hasi

16. Ni mara ngapi wewe hupima VVU?

☐1 = mara moja katika mwaka ☐2= mara 1 au 2 kwa mwaka

☐3= Mara 3 au 4 katika mwaka ☐4= Zaidi ya mara 4 katika mwaka

D. Tabia zinazohusiana na hatari za kuambukizwa na VVU

17. Umekuwa na wapenzi wangapi katika miezi 6 iliyopita? (Kama una uhakika, tafadhali kadiria) |___|___|

18. Kwa sasa, unao wapenzi wangapi? |___|___|

19. Je, Unafahamu hali ya VVU ya mpenzi wako? ☐1= Ndio ☐2= La ☐3= Sijui

20. Kama ndio kwa swali la (20), nini hali ya VVU ya mpenzi wako? ☐1= Chanya ☐2= Hasi

21. Je, wewe hutumia mipira ya kondomu kila wakati unapo jamiiana? ☐1= Ndio

    ☐2= La ☐3= Wakati mwingine

22. Umewahi kushiriki katika ngono kwa ajili ya fedha, nyumba, dawa, ulinzi, au bidhaa nyinginezo? ☐1=Ndio ☐2=La

E. <u>Kunywa pombe na kutumia madawa ya kulevya</u>

23. Ni mara ngapi wewe kunywa pombe? *(If No skip to29)*

    ☐1 = Sijawahi kunywa pombe ☐2= mara moja kwa wiki

    ☐3= Mara 2-4 kwa wiki ☐4= kila siku ya wiki ☐5= Mara moja kwa mwezi

24. Ni aina gani ya pombe wewe hukunya?

    ☐1= Mvinyo ☐2= Bia

    ☐3= Spirits/Waragi/gin/whisky/vodka ☐4=Pombe ya mitaa (taja)

25. Ni kwa wakati gani kwa siku ambapo wewe kunywa pombe?

    ☐1= Mapema asubuhi ☐2= Wakati wa chamcha

    ☐3= Jioni baada ya kazi ☐4= Siku nzima nifanyapo kazi

27. Je, Unayo uwezekano Zaidi ya kushiriki katika ngono baada ya kunywa pombe?

    ☐1= Ndio ☐2= La ☐3= Sijui

28. Hata baada ya kunywa pombe, wewe bado hutumia mpira wa kondomu?

☐1=Ndio ☐2=La

29. Je, unatumia dawa yoyote ya kulevya? ☐1=Ndio ☐2=La *(If No skip to Qn.32)*

30. Ni dawa ya kulevya ipi ambayo wewe hutumia?

☐1 = Bangi / Shisha ☐2= Miraa ☐3= Petroli

☐4= Kubba ☐5= Nyinginezo (taja) ___________

31. Ni mara ngapi wewe hutumia madawa ya kulevya?

☐1 = Mara moja kwa wiki ☐2= mara 2-4 kwa wiki

☐3= kila siku kwa wiki ☐4= Mara moja kwa mwezi

F. Maoni juu ya kupimo cha vvu kutumia sampuli ya mdomo

32. Je, umewahi kusikia kuhusu kipimo cha VVU cha upesi ya kutumia sampuli za mdomo?

☐1=Ndio ☐2=La

33. Je, utaweza kutathmini kutumia kipimo cha VVU cha upesi ya kutumia sampuli za mdomo?

☐1=Ndio ☐2=La

34. Je, utapendelea kutumia kipimo cha VVU cha upesi ya kutumia sampuli za mdomo ikiwa itakuwa inapatikana ulipo? ☐1= Ndiyo ☐2= La ☐3= Sijui

35. Ni kwa sababu zipi ambazo utapendelea kutumia kipimo cha VVU cha upesi ya kutumia sampuli za mdomo? ***(Chagua zote zilizo sahihi kwako)***

☐1= Hakuna uchungu kwa mbinu hii ☐2= Hakuna damu inayotolewa

☐3= Muda mfupi kabla ya matokeo ☐4= Inatoa nafasi nzuri ya usiri

☐5= hakuna kudungwa ☐6= Ni ya kuaminika ☐7= Urahisi wa kukusanya sampuli

☐8= Uraisi ☐9= Naweza kujipima kibinafsi mara kadha

☐ 10= Nyinginezo (taja) ___________

36. Ni sababu zipi ambazo zitakufanya kutokubali kutumia kipimo cha VVU cha upesi ya kutumia sampuli za mdomo? ***(Chagua zote zilizo sahihi kwako)***

☐ 1= Sijawahi skia juu ya kipimo hiki ☐ 2= Sina uhakika kuhusu usahihi wake

☐ 3= Napendelea kipimo cha damu ☐ 4= Sitaki kutumia mbinu mpya

☐ 5= Nina uhakika zaidi kama nimepimwa kutumia damu ☐ 6= Haina uhakika

☐ 7= Nyinginezo (taja) ___________

Matokeo ya vipimo ya maabara *(kujazwa na mshauri)*

37. Hali ya kuambukizwa kwa damu baada ya kipimo upesi cha VVU kutumia sampuli ya damu

☐ 1= Chanya ☐ 2= Hasi

38. Hali ya kuambukizwa kwa damu baada ya kipimo cha VVU cha upesi ya kutumia sampuli za mdomo? ☐ 1= Chanya ☐ 2= Hasi

39. Je, majibu haya yanakubaliana? ☐ 1= Ndio ☐ 2= La

(To be filled only after repeat testing on the serial blood algorithm)

40. Ni matokeo ya kupima VVU ni nini?

☐ 1= Hasi ☐ 2= Chanya
