# Supplementary material for: Diagnostic accuracy and acceptability of rapid HIV oral testing among adults attending an urban public health facility in Kampala, Uganda
Source: PLoS One. 2017 Aug 23;12(8):e0182050. doi: 10.1371/journal.pone.0182050 (PMC5568333; doi:10.1371/journal.pone.0182050)
Supplement: S2 File — (PDF) [file pone.0182050.s002.pdf]

Topic guide for the Focus Group Discussion in English

Date (dd/mm/yyyy) ___________________

Moderator ________________________

Recorder __________________________

Language used ________________________

Time: Start _________________ End _____________________

Good morning/afternoon

You are welcome to this discussion. My name is _________________________ and my colleague (recorder) is _______________________

We are from the Makerere University College of Health Sciences and we would like to have a chat about your opinions on the HIV oral fluid test and issues related to its acceptability (willingness Vs unwillingness to use it). The purpose of this discussion is to get your views and then make appropriate recommendations to health care providers and MOH regarding introduction of the HIV oral fluid test as a method for testing HIV infection. We are not in any way connected to the management of KCCA health centres so feel free to express yourself and just be honest. Remember there is no wrong opinion. We also have a tape recorder to help us remember this discussion, may we use it? ***(Moderator asks consent).***

Thank you very much.

In order to ease the conversation, we would like to first get to know each other's first name? ***(One name only).***

1. In Uganda, we test for HIV using only blood which is obtained either by finger prick or venepuncture. What do you think about this method in testing for HIV infection?
2. According to you, are there any challenges related to use of blood specimens when testing for HIV?
3. HIV testing methods have advanced to incorporate use of other specimens like saliva. Do you know anything concerning how saliva is used to test for HIV?
4. Have you ever heard about the HIV oral fluid test? What do you know about it?
5. In this study, we tested some clients perhaps even some of you using the HIV oral fluid test, Could you share with us your experience of using this test?
6. Do you think people's HIV testing behaviours will be influenced if the HIV oral fluid test is introduced in Uganda?
7. In future, would you ever consider testing for HIV infection using the HIV oral fluid test if it is available?
8. Why would you choose to use or not use the HIV oral fluid test?

Topic guide for the Focus Group Discussion in Luganda

Ennaku z'omwezi (dd/mm/yyyy) ____________________

Omuwuwutanyi _________________________

Omuwaabi ___________________________

Olulimi olukozeseddwa _________________________

Enkozesa y'obuudde: Okutandika _____________ Okumaliriza______________________

Mwasuzze mutya/Mwasiibye mutya Bassebo n'ebannyabo

Tubanirizanyo mukuteesa kuno leero. Nze ________________ ne munange ye ________________________

Tuviridde musetendekero e'Makerere University ku college of Health Sciences mu kitongole ekinonyereza ku ndwade. Twagala kuteesa n'okukubaganya ebirowooza ku ngeli yo kukebera akawuuka kamukenenya nga tukozesa amaluusu. Okusingiradala ebirozowoozo ebikwatagaana n'okukirirza/okuwagira oba okuwakanya/okugana enkola eno mukukebera akawuuka kamukenenya. Ebiroowoozo byamwe kunkola eno bijja kuyamba muntekateka zebyobula m'Uganda naddala kungeli yo kwanjula mu technologia ow'okukozesa amaluusu mukukebera akawuuka kamukenenya. Ffe tetulina nkolagana yanjawulo yona n'akaiiko akafuga amalwariro ga KCCA era tubasaba mubere neddembe elyokuteesa era mwogere amazima. N'ensonga endala gye mwetaaga okumanya eri nti tewaliwo kiteeso kikyamu n'olwekyo bulyomu asobola okuwa ekiteso kye. Wabula ekilala twazze n'olukoba olukwata amaloobozi era tusaba mutukirize okulukozesa okukwata n'okutereka emboozi eno. ***(Omuwuwutanyi yaja okusaba olukusa).***

Mwebale nyo.

Mungeli y'okwanguya enzirukanya y'okuteesa kuno, tujja kutandika n'okweyanjula nga twogera amanya gaffe naddala errinya lye ddini tusobole okwemanya awo tusobole okuteesa bulungi.

1. M'uganda, tukebera akawuuka kamukenenya nga tukozesa omusaayi. Omusaayi gufunibwa mukufumita engalo oba kumukono. Mulowooza ki kunkola eno ey'okukebera akawuuka kamukenenya?
2. Okusinzira kumwe, mulowooza waliwo ebintu byonna ebisoomooza mukukozesa omussayi okukebera akawuuka kamukenenya?
3. Engeli z'okukeberamu akawuuka kamunenya zikyuuse nezeyoongera omutindo era nga kati waliwo n'engeli eyokukozesa amaluusu mukukebera silimu. Ani ayiina kyamanyi kungeli yokukozesa amaluusu mukukebera akawuuka kamukenenya?
4. Mu kunonyereza kuno, tukebedde abantu abamu sinakindi abamu kubakeberebwa akawuuka kamukenenya nga tukozesa amaluusu muliwano, Waliwo asobola okutubuuliirako kungeli gyeyawulira oba gyeyasanga mu okukebera akawuuka kamukenenya ngatukozesa amaluusu?
5. Singa engeli y'okukebera akawuuka kamukenenya nga tukozesa amaluusu eleteebwa m'Uganda, mulowooza kiyinza okukyusa obujumbizze oba okuziyiza abantu okwekebeza mukenenya?
6. Gye bujja mumaaso, mulowooza musobola okwekebeza akawuuka kamukenenya ngamukozesa amaluusu singa enkola eno eb'etekeedwawo?
7. Mulowoza nsonga ki eziyinza okulobera okukozesa oba obutakozesa malusu nga wekebeza akawuuka kamukenenya?

Topic guide for the Focus Group Discussion in Kiswahili

KIELEKEZO CHA MAJADILIANO YA VIKUNDI

Tarehe: (dd/mm/yyyy) ___________________

Msimamizi: ________________________

Karani/katibu: __________________________

Lugha inayotumika: ________________________

Saa: Kuanza _________________ Tamati ____________________

Habari ya asubuhi/Mchana

Mnakaribishwa katika mjadala huu. Jina langu ni _________________________ na mwenzangu (karani) ni _______________________

Twatoka katika Chuo Kikuu cha Makerere (Chuo cha Sayansi ya Afya). Tungependa kuwa na mazungumzo kuhusu maoni yenu juu ya kipimo cha VVU kutumia njia ya sampuli za mdomo. Pia, tungependa kufahamu masuala yanayohusiana na kukubalika au kutokubalika kwake. Lengo la mjadala huu ni kupata maoni yenu na kisha kutoa mapendekezo sahihi kwa watoa huduma za afya na Wizara ya afya kwa ajili ya kuanzishwa kwa kipimo cha VVU kutumia sampuli za mdomo. Hatuna uhusiano wowote na kituo cha afya ya KCCA kwa hivyo tungependelea mjiskie huru kujieleza kwa kwa ukweli. Tunge penda kuwahimiza ya kuwa hakuna maoni isiyo na thamani. Tunayo chombo cha kunasa sauti za mjadala kutusaidia kukumbuka mazungumzo haya. Tunataka kuomba ruhusa ya kutumia chombo hiki cha kunasa sauti? *(Msimamizi ataomba idhini).*

Asanteni sana.

Kwanza, tungependa kujua jina la kila mtu aliye hapa kabla ya kuanza mazungumzo *(Jina la kwanza).*

1. Humu nchini Uganda, desturi ni kuwa kwa kupima hali ya VVU; damu hutolowe kwenye kidole kwa kudungwa au kwenye mishipa ya mkono. Je, maoni yako ni yepi kuhusu njia hii ya kupima kiambukizo cha VVU?
2. Kwa mujibu wako, je, kuna changamoto zozote kutokana na matumizi ya sampuli ya damu wakati wa kupima VVU?
3. Mbinu za kupima VVU vimeboreshwa kujumuisha sampuli zingine kama mate. Je, wafahamu chochote kuhusu jinsi mate hutumika kupima VVU?
4. Je, umewahi kusikia kuhusu kipimo cha VVU kwa kutumia sampuli za mdomo? Wafamu nini kuhusu jambo hili?
5. Katika utafiti huu, tulipata kupima baadhi ya washirika hali yao ya VVU na pengine hata baadhi yenu kwa kutumia sampuli za mdomo, Je, mnaweza kutusimulia hisia au fikra zenu kuhusu matumizi ya mbinu hii ya kipimo?
6. Je, unafikiri tabia za watu kuhusu kutaka kupimwa hali yao ya VVU itabadilishwa ikiwa mbinu hii italetwa na kutumika humu nchini Uganda?
7. Katika siku zijazo, unaweza kupendelea kupimwa hali ya kuambukizwa na VVU kwa kutumia mbinu hii ya sampuli za mdomo ikiwa itakuwepo?
8. Toa sababu ambazo zitachangia wewe kuchagua au kutochagua kutumia sampuli za mdomo kujua hali yako ka kimbukizo cha VVU?
